# Influence of Bagging on Fruit Quality, Incidence of Peel Browning Spots, and Lignin Content of ‘Huangguan’ Pears

**DOI:** 10.3390/plants13040516

**Published:** 2024-02-13

**Authors:** Yeqing Guan, Xiaoli Qin, Chuangqi Wei, Yunxiao Feng, Yudou Cheng, Yang Zhang, Junfeng Guan

**Affiliations:** Institute of Biotechnology and Food Science, Hebei Academy of Agriculture and Forestry Sciences, Shijiazhuang 050051, China; guanyeqing@126.com (Y.G.); 15903628870@163.com (X.Q.); weichuangqi@163.com (C.W.); fengyunxiao88@163.com (Y.F.); chengyudouyn@163.com (Y.C.); taloyament@163.com (Y.Z.)

**Keywords:** Huangguan, bagging, fruit surface, peel browning spots, lignin

## Abstract

The ‘Huangguan’ pear is one of the high-quality pear cultivars produced in China. However, the bagged fruit of the ‘Huangguan’ pear often suffers from peel browning spots after rain during their mature period. In this study, in an effort to discover the impact of bagging treatments on the occurrence of peel browning spots and fruit quality, fruits were covered by single-layer, two-layer, or triple-layer paper bags six weeks after reaching full bloom. The results showed that the bagged fruits were characterized by smooth surfaces and reduced lenticels compared with the unbagged ones. The unbagged and the two-layer bagged fruits had yellow/green peels, while the single- and triple-layer bagged ones had yellow/white peels. Compared with the unbagged fruits, the bagged fruits had higher vitamin C (Vc) contents and values of peel color indexes L and a and lower soluble solid contents (SSCs), titratable acid (TA) contents, absorbance index differences (I_AD_), and b values. Additionally, the triple-layer bagged group was superior to other groups in terms of fruit quality, but it also had the maximum incidence of peel browning spots. Before and after the appearance of peel browning spots, the bagged fruits had smoother and thinner cuticles compared with the unbagged ones. Furthermore, the triple-layer bagged fruits had minimum lignin contents and maximum phenolic contents in their peels, with minimum activity of lignin synthesis-related enzymes such as phenylalanine ammonia lyase (PAL), peroxidase (POD), and polyphenol oxidase (PPO), as well as minimum expressions of relevant genes such as cinnamyl alcohol dehydrogenase (*CAD*), cinnamoyl CoA reductase (*CCR*), 4-coumarate: coenzyme A ligase (*4CL6*), and cinnamate 4-hydroxylase (*C4H1*). It was deduced that POD activity and the relative expressions of *CAD9*, *CCR3*, *CCR4,* and *CCR5* may play key roles in the occurrence of peel browning spots. In summary, lignin synthesis affected the incidence of peel browning spots in bagged ‘Huangguan’ pears. This study provides a theoretical basis for understanding the incidence of peel browning spots in ‘Huangguan’ pears.

## 1. Introduction

The ‘Huangguan’ pear (*Pyrus bretschneideri* Rehd) is a high-quality pear variety that has experienced rapid growth in popularity in China. Indeed, due to its appealing shape, tender flesh, and exceptional quality, it has gained widespread consumer appreciation, hence indicating promise for further development.

An essential practice for producing high-quality pears is bagging, a protective measure that helps to modify the peel color, improve the fruit’s smoothness, reduce lenticels, and optimize the peel’s tissue structure. This technique also goes beyond enhancing the fruit’s visual appearance by impacting the fruit’s firmness, soluble solid contents (SSCs), and volatile substance contents to varying degrees [1,2,3,4]. However, despite its advantages, bagging can be particularly challenging during the ripening season of Huangguan pears due to peel browning spots (commonly known as “chicken foot disease”), which are likely to occur on the surface of bagged pears. This browning process, which tends to occur rapidly, significantly undermines the commercial value of the fruit [5].

In pears, phenolics are one of the main secondary metabolites that influence both fruit quality and browning [6,7]. Lignin, a polymer of phenolic compounds, is involved in the formation of lenticels and is synthesized through the phenylpropanoid metabolic pathways. The key enzymes in this process include phenylalanine ammonia lyase (PAL), cinnamate 4-hydroxylase (C4H), 4-coumarate: coenzyme A ligase (4CL), cinnamoyl CoA reductase (CCR) and cinnamyl alcohol dehydrogenase (CAD), which produce lignin monomers that are subsequently converted into lignin in the presence of peroxidase (POD) and polyphenol oxidase (PPO) [8]. Given the involvement of these enzymes, their gene expression is therefore closely linked to lignin synthesis [9,10,11,12,13].

The aim of this study was to elucidate the impact of different bagging treatments on the appearance, fruit quality, activities of lignin synthesis-related enzymes, and gene expressions related to lignin synthesis in ‘Huangguan’ pears. It is expected that the findings will help to explain the reasons behind peel browning spots in ‘Huangguan’ pears and provide a theoretical basis for understanding and addressing its incidence.

## 2. Results

### 2.1. Effects of Bagging on the Phenotype and Quality Development of ‘Huangguan’ Pears

**Figure 1:**
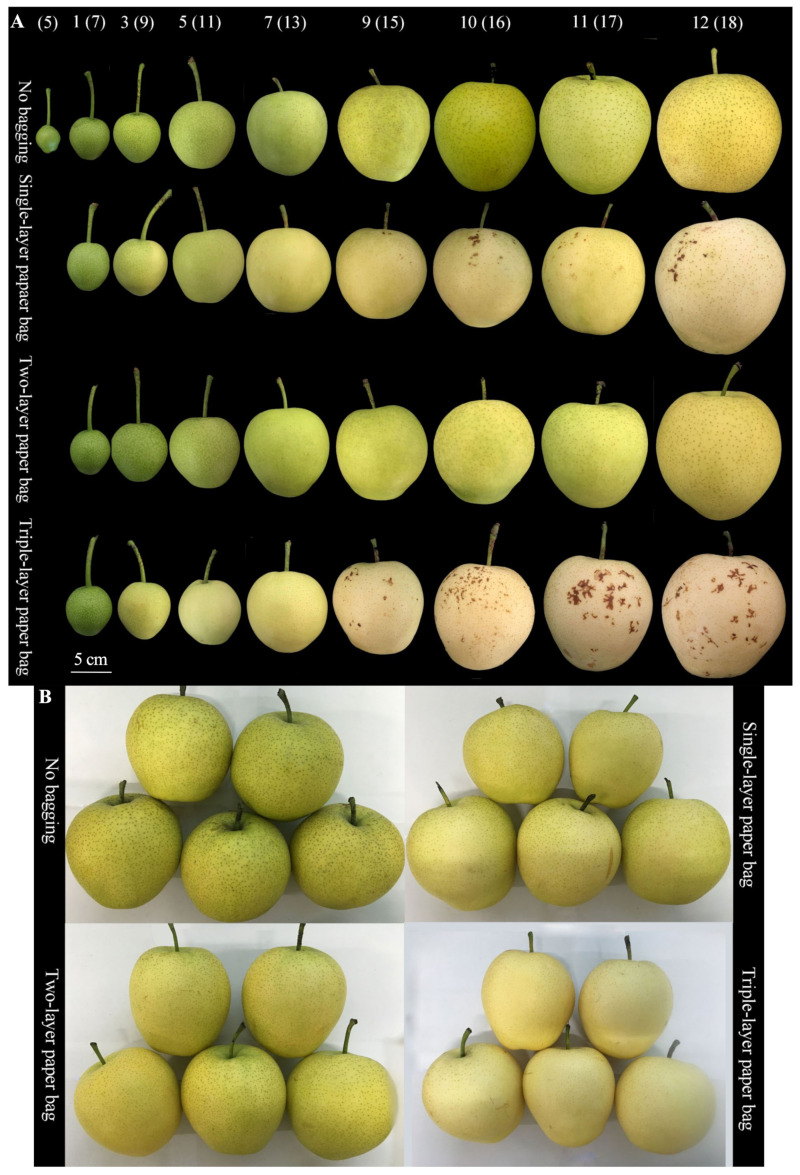
Phenotypic observation of the effect of bagging on the fruit of ‘Huangguan’ pears at different development periods. Growth period (**A**). Healthy fruit at harvest time (**B**). Numbers indicate weeks after bagging (weeks after full bloom); the same below.

As the ‘Huangguan’ pears matured, there was a noticeable increase in their volume, and their colors changed from green to yellow (Figure 1A). For unbagged and two-layer bagged fruits, the colors shifted from green to yellow-green. In contrast, fruits in single- and triple-layer bags changed from green to yellow-white and even displayed earlier color transformations. Furthermore, compared with unbagged fruits, the bagged ones exhibited cleaner and smoother surfaces, with fewer and smaller lenticels. However, after nine weeks of bagging, peel browning spots emerged. These were particularly pronounced on the surfaces of single- and triple-layer bagged fruits, with the latter having the largest areas of peel browning spots. On the other hand, the two-layer bagged fruits showed fewer peel browning spots, while the unbagged ones did not exhibit peel browning spots. Upon ripening, the bagged fruits displayed smooth surfaces, while the unbagged ones had rough surfaces with dust attached. In fact, the latter’s colors were also darker than those of the two-layer bagged fruits, which instead exhibited yellowish green hues. In contrast, single- and triple-layer bagged fruits were yellowish white. Finally, the bagged fruits exhibited smaller and lighter-colored lenticels, with fewer lenticels per unit area (Figure 1B).

**Figure 2:**
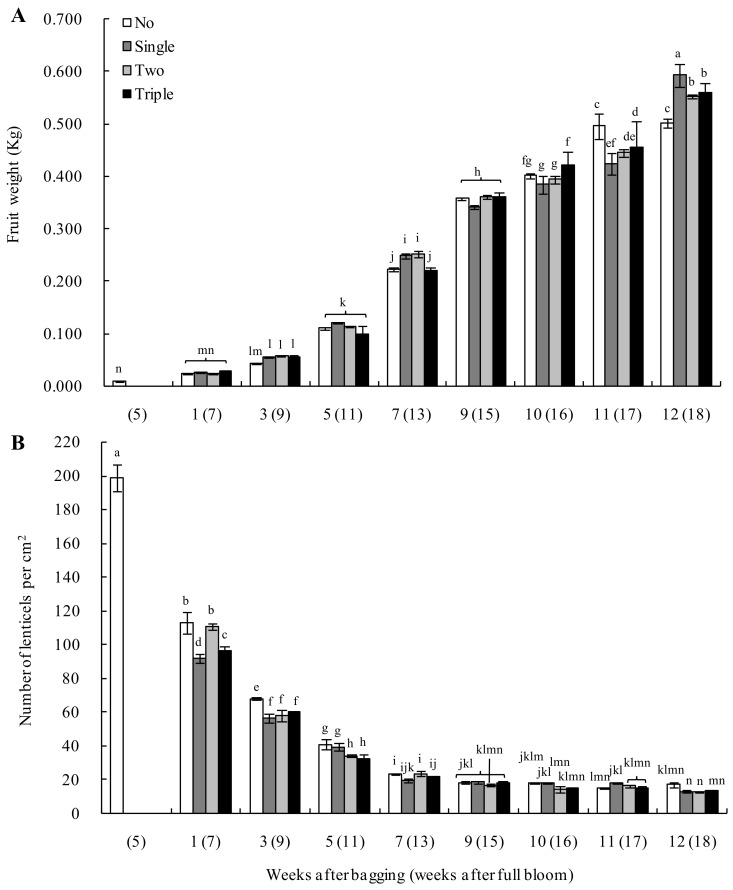
Effect of bagging on the fruit weight (**A**) and number of lenticels per cm^2^ (**B**) during development of ‘Huangguan’ pears. Different lowercase letters indicate significant differences (*p* < 0.05).

As the fruit matured, the fruit weight of all bagged groups showed an upward trend. Meanwhile, for unbagged ones, the fruit weight was lower than that obtained after bagging for one to seven weeks. In addition, after bagging for 10 weeks, the different bagged groups did not differ significantly in terms of fruit weight. However, there were differences in fruit weight among the different bagged groups, although the pattern was not distinct during 11–12 weeks after bagging (Figure 2A). Regarding lenticels, bagging for one to five weeks significantly decreased the number of lenticels on the surface of different bagged fruits, while unbagged ones had a significantly higher number than the bagged groups. However, after bagging for seven weeks, the rate of decline in the number of lenticels leveled off, showing no significant differences between the groups (Figure 2B).

**Figure 3:**
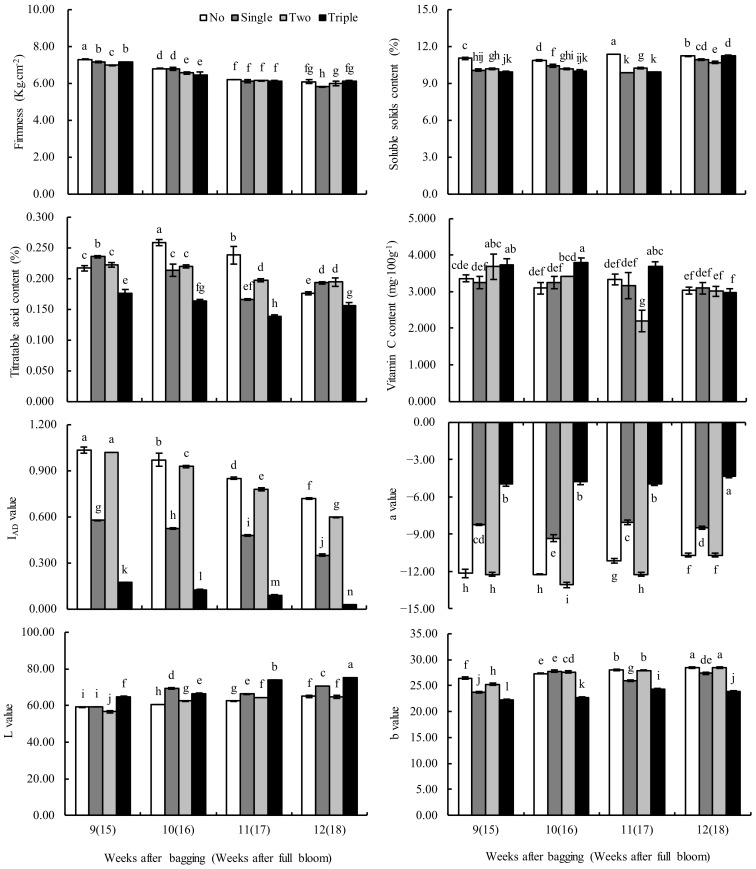
Effect of bagging on the fruit quality of ‘Huangguan’ pears during the mature period. Different lowercase letters indicate significant differences (*p* < 0.05).

As shown in Figure 3, as maturity approached (after bagging for 9–12 W), fruit firmness, titratable acid (TA), vitamin C (Vc) and absorbance index differences (I_AD_) of different bagged groups showed a decreasing trend, while SSCs and peel color indexes L, a and b continued to rise. Regarding the peel color index, the unbagged and two-layer bagged fruits exhibited a consistent trend, similar to that shared between single-and triple-layer bagged fruits. In general, except for firmness, the Vc contents as well as the L values and a values of different bagged groups were higher than those of unbagged fruits. In contrast, the SSCs, TA contents, I_AD_ values and b values were lower than those of the unbagged fruits, especially in the case of the triple-layer bag. The results suggested that the fruit quality of triple-layer bagged fruits was generally better than that of single- and two-layer bagged or unbagged fruits.

### 2.2. Effects of Bagging on the Incidence of Peel Browning Spots and Structure of ‘Huangguan’ Pears

After bagging for seven weeks, there were no peel browning spots of different bagged fruits. However, peel browning spots appeared on the fruits of different paper bags after nine weeks of bagging, with the highest incidence for triple-layer bagged fruits. The incidence of two-layer bagged fruits was relatively low, and no disease occurred for the unbagged fruits (Table 1). In order to further reveal the reason for the occurrence of peel browning spots, the following study focuses on the stage of seven to nine weeks after bagging (the critical period of the occurrence of the peel browning spots).

**Figure 4:**
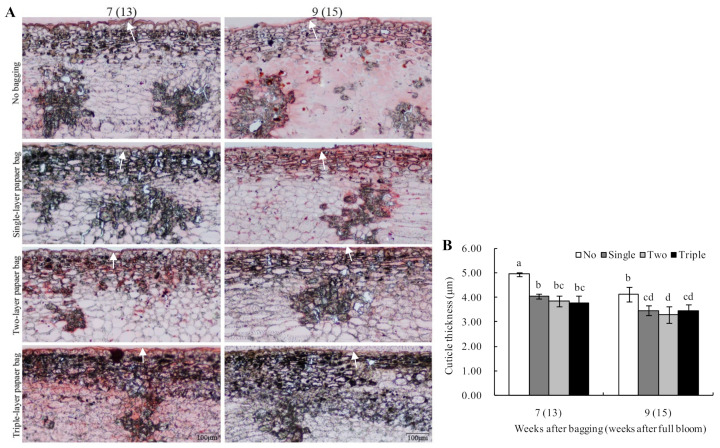
Effect of bagging on cuticle structure (**A**) and thickness (**B**) of peel in ‘Huangguan’ pears. Cuticles are indicated with white arrows. Different lowercase letters indicate significant differences (*p* < 0.05).

As shown in Figure 4A, the cuticles of unbagged fruits were smooth and fluctuated alongside the epidermal cells after bagging for seven to nine weeks. As the fruit grew and developed, the epidermal cells under the stratum corneum of different bagged groups increased in volume, were arranged loosely and had gaps between cells.

Based on the statistics, the cuticles’ thicknesses of different bagged fruits decreased during this period. Compared with the unbagged fruits, the cuticles’ thicknesses of the bagged fruits decreased significantly after seven to nine weeks of bagging. Although the cuticles’ thicknesses of single-, two-layer and triple-layer bagged fruit differed, the differences were not noticeable (Figure 4B).

**Figure 5:**
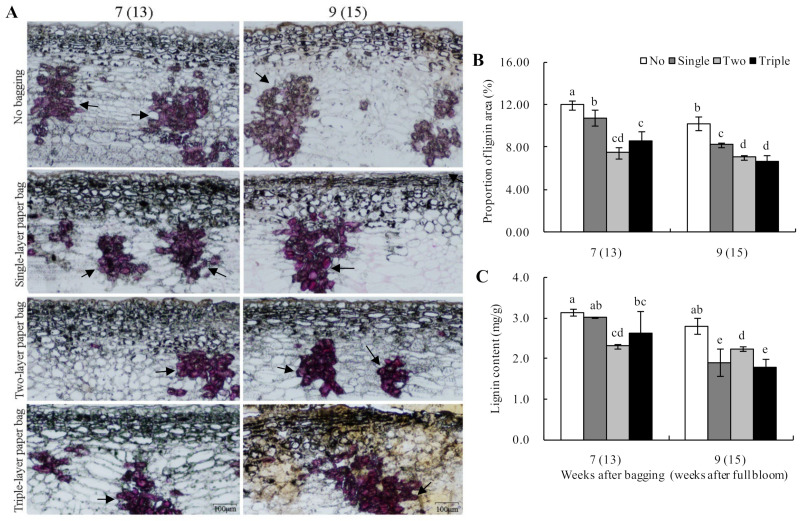
Effect of bagging on lignin distribution (**A**) and contents (**B**,**C**) of ‘Huangguan’ pears. Dyed lignin is indicated with black arrows; the proportion of lignin area = lignin area in visual field/peel area in visual field. Different lowercase letters indicate significant differences (*p* < 0.05).

As shown in Figure 5A,B, the peel lignin of the ‘Huangguan’ pears was mainly distributed in the cork layer, and lignin was irregularly distributed in the peel. After bagging for seven to nine weeks, the lignin distribution areas of the unbagged and bagged fruits continued to decrease. The lignin distribution areas of the bagged groups were also significantly smaller than that of the unbagged fruits after bagging for nine weeks, with the area for triple-layer bagged fruits being the smallest. After bagging for nine weeks, a yellow area was observed in the triple-layer bagged fruits, which was presumed to be a brown-spotted area.

At the same time, lignin contents were measured, and after seven to nine weeks of bagging, a downward trend was noted for the different bagged groups. The lignin contents in the bagged groups were significantly lower than that of the unbagged fruits. At nine weeks (appearance of peel browning spots), the lignin contents of single- and triple-layer bagged fruits were the lowest (Figure 5C). Additionally, a significant correlation was noted between lignin distribution and content (r = 0.899, *p* < 0.01), indicating a consistent trend in lignin distribution and content.

### 2.3. Effects of Bagging on the Incidence of Peel Browning Spots, Total Phenolic Contents and Activities of Related Enzymes in ‘Huangguan’ Pears

**Figure 6:**
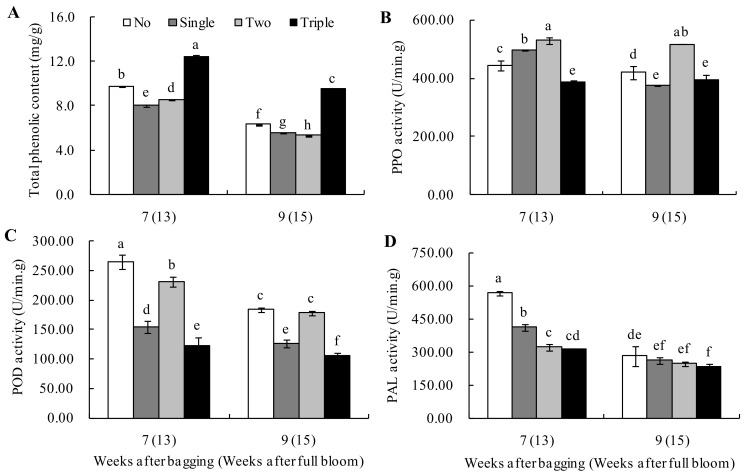
Effect of bagging on total phenolic contents (**A**), PPO (**B**), POD (**C**) and PAL (**D**) activities of peels in ‘Huangguan’ pears. Different lowercase letters indicate significant differences (*p* < 0.05).

The total phenolic contents and activities of related enzymes in different bagged groups decreased after bagging for seven to nine weeks (Figure 6). The total phenolic contents in the peel of triple-layer bagged fruits were significantly higher than that of other bagged groups (Figure 6A). Furthermore, the unbagged and two-layer bagged fruits had substantially higher PPO and POD activities than those of single- and triple-layer bagged fruits. PPO and POD activities were the lowest in triple-layer bagged fruits, while for two-layer bagged fruits, the PPO activity was the highest (Figure 6B). In addition, the POD activity for unbagged fruits was the highest (Figure 6C). The PAL activity of the unbagged fruits was higher than that of the bagged groups and the activity of the triple-layer bagged fruits was significantly lower than that of the unbagged fruits (Figure 6D).

### 2.4. Effects of Bagging on the Expressions of Genes Related to the Incidence of Peel Browning Spots in ‘Huangguan’ Pears

**Figure 7:**
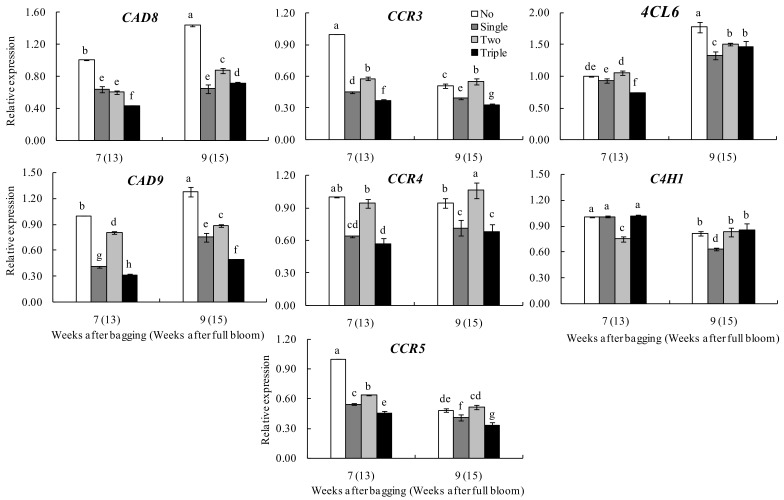
Effect of bagging on the expression of genes related to peel browning in ‘Huangguan’ pears. Reference gene: Actin2; *n* = 3. Different lowercase letters indicate significant differences (*p* < 0.05).

As shown in Figure 7, bagging for seven to nine weeks increased the relative expressions of *CAD8* and *CAD9* in the peels of all bagged groups, with maximum expression achieved after bagging for nine weeks (appearance of peel browning spots). In general, the expression levels of these two genes in unbagged fruits and two-layer bagged fruits were significantly higher than those in single- and triple-layer bagged fruits at weeks seven to nine of bagging.

After bagging for seven to nine weeks, the relative expressions of *CCR3* and *CCR5* in the peels of different bagged groups gradually decreased, except for *CCR4*. The relative expressions of *CCR3*, *CCR4* and *CCR5* were also significantly higher in the peels of unbagged and two-layer bagged fruits than in single- and triple-layer bagged fruits.

The relative expression of the *4CL6* gene in the peels of all bagged groups increased after bagging for seven to nine weeks. However, beyond nine weeks of bagging, the relative expressions of the genes in the bagged groups were significantly lower compared with the unbagged fruits. In addition, the expressions in two-layer bagged fruits were higher than that in single- or triple-layer bagged ones, but the differences were insignificant.

After bagging for seven to nine weeks, the relative expression of the *C4H1* gene in the peels of two-layer bagged fruits increased while those in the peels from other groups decreased. Thus, no specific trend was noted for the different bagged groups.

## 3. Discussion

### 3.1. Changes in Fruit Development

Bagging is a physical protection measure widely used in a variety of fruit as it can improve the appearance of fruit by promoting a change in fruit color and reducing the production of microcracks and rust on the fruit surface [14]. In this study, during the growth and development of the ‘Huangguan’ pear, the bagged fruits exhibited a distinctive appearance with a smooth and polished surface, characterized by reduced lenticels compared to the unbagged fruits. There was a considerable decrease in the density of lenticels per unit area of bagged fruits throughout the initial phases (one to five weeks after bagging). The fruit colors of the unbagged fruits and two-layer bagged fruits were similar, changing from green to yellow-green. Single- and triple-layer bagged fruit also had similarities, when changing from green to yellow-white. When ‘Huangguan’ pears matured, the peel color parameters such as L and a in the bagged fruits were significantly higher than the unbagged ones. At the same time, I_AD_ and b values were significantly lower. The changes in peel color parameters in different bagged groups matched the phenotypic color changes in fruits. It has been demonstrated that ‘Cuiguan’ and ‘Housui’ pear fruits with bagging had more brightness, less rust and lenticels than the unbagged fruits, which improved their market value [15,16]. The ‘sand pear’ peels were light yellow after bagging, consistent with the ‘Concorde’ pear after bagging. Compared with the control, values of the L, h^0^ and a of ‘sand pear’ were higher, while the fruit firmness increased, and the SSCs decreased [3,17]. Similarly, Feng et al. [18] found that bagged ‘Jonagold’ apple fruit were yellowish and had higher lightness and hue angles than the control. Also, fruit firmness was slightly increased, whereas SSCs decreased.

In this study, when ‘Huangguan’ pears matured, the overall SSC and TA contents were significantly lower than the unbagged ones, while the Vc contents were significantly higher. The triple-layer bagged fruit’s quality was better than the other treatments. Bagging can change the microenvironment of fruit development and have multiple effects on the internal quality of fruit. For example, bagging could significantly increase (e.g., pomegranate), decrease (e.g., guava), or do not affect (e.g., pear) SSCs [14]. Similarly, previous results have reported that after bagging, the sugar–acid ratio and Vc contents of ‘Huangguan’ pears were higher [19]. Again, the chlorophyll contents of the peels were significantly reduced [20], while the acid concentration of bagged apples was also reduced [21]. Furthermore, throughout the growth period, the fruit weight of each fruit grew in the various bagged groups, according to this study. However, there was no discernible pattern between the unbagged and the bagged fruits. Likewise, Sharma et al. [4] found that bagging can increase (e.g., carambola, mango, longan), reduce (e.g., loquat, pear, pomegranate, apple) or have no effect (e.g., banana, pear) on fruit weight.

### 3.2. Mechanism of Peel Browning Spots

In this study, peel browning spots were observed in the ninth week after bagging, and the incidence of the peel browning spots in the triple-layer bagged fruits was significantly higher compared with other groups. In contrast, fruits that were bagged in two layers showed the least occurrence of peel browning spots, whereas unbagged fruits did not show any spots. After seven to nine weeks of bagging, the unbagged fruits exhibited an irregular texture on the outer layer of epidermal cells. Still, the bagged fruits had a smooth cuticular layer with a significantly reduced thickness compared to the unbagged fruit. Wang et al. [1] also found that the incidence of peel browning spots was significantly higher in the three-layer bagged fruits than in non-bagged ones of ‘Huangguan’ pears. Moreover, the cuticle of unbagged fruits pear was thick and wavy, while the cuticle of bagged fruits was uniform and significantly thinner than that of unbagged fruits. Likewise, the cuticle became thinner after bagging in pears and apples [2,22]. Wang et al. [23] reported that the lignin contents decreased drastically after bagging in ‘Dangshan’ pears. In this study, after seven to nine weeks of bagging, the lignin contents in bagged fruits were considerably lower than in unbagged ones, and the distribution area was smaller. It suggested that a reduced lignin content may be associated with the formation of peel browning spots.

Previous reports proved that bagging could significantly reduce the phenolic contents in the peels of pears and apples [3,18,24]. During the early growth stage, Guan et al. [25] found that bagging could reduce the phenolic contents in the ‘Huangguan’ pear peels. However, in this study, after bagging for seven to nine weeks, the phenolic contents in the peels of single-layer and two-layer bagged ‘Huangguan’ pears were lower than that of unbagged fruit, although surprisingly, the phenolic contents in the peels of triple-layer bagged fruits were significantly higher than the others. This could be related to phenol accumulation caused by significant peel browning spots on triple-layer bagged fruits. Similarly, Chen et al. [24] found that polyphenols in plants are connected with poor environmental conditions. Indeed, bagging reduced the activity of phenol metabolic-related enzymes in apple fruits [26,27]. PAL, a key enzyme in the process of phenylpropane metabolism to lignin production, is induced by light [28]. Its activity was also inhibited by bagging throughout the growth period in the ‘Dangshan’ pear fruits [24]. PPO and POD activity are the main factors leading to browning disorders [29,30]. Jiang et al. [31] discovered that bagging of pear fruits inhibited the enzyme activities of PAL and POD. Meanwhile, Zhang et al. [22] demonstrated that PPO and POD activities decreased after bagging ‘Laiyang’ pears. In this study, the fruits were bagged for seven to nine weeks. The activities of POD and PAL in bagged fruits were lower than that of unbagged fruits, with the lowest value recorded in triple-layer bagged fruits. In addition, the PPO activity was significantly lower in triple-layer bagged fruit than in the others. It was also found that as the PPO, POD and PAL activities were inhibited, lignin biosynthesis was hindered in the post-harvest senescence browning of bamboo shoots [32]. Our study also found that the changing trend of POD activity of unbagged fruits was consistent with that of two-layer bagged fruits. Meanwhile, the changing trend of POD activity of single-layer bagged fruits was consistent with that of triple-layer bagged ones. As mentioned above, the incidences of peel browning spots of single- and triple-layer bagged fruits were more severe than that of unbagged and two-layer bagged fruits. Hence, POD activity could be essential in peel browning spots. Heng et al. [33] discovered that POD plays a critical role in the lignin biosynthesis process, catalyzing the polymerization of lignin monomers to create lignin. As a result, future research should focus on how light affects POD in the lignin production pathway to regulate the occurrence of browning.

Ju et al. [26,27], Takos et al. [34], and Yu et al. [35] demonstrated that, in unbagged apples and pears, the activities/expressions of key enzymes/genes in the phenol metabolism pathways were up-regulated after light exposure. The activities of crucial lignin synthesis-related enzymes C4H, 4CL and CAD in the developmental fruit of ‘Dangshan’ pears were inhibited after bagging [23]. Similarly, in other fruits, the expression of *C4H*, *4CL*, *CCR* and *CAD* genes was positively correlated with lignin synthesis [9,10,11,13]. This study observed that the *CAD* and *4CL6* genes related to lignin synthesis in ‘Huangguan’ pears were significantly up-regulated. At the same time, *CCR3*, *CCR5* and *C4H1* were down-regulated considerably after seven to nine weeks of bagging. Specifically, the expression levels of *CAD9*, *CCR3*, *CCR4* and *CCR5* in the unbagged and two-layer bagged fruits were significantly higher than those in the single- and triple-layer bagged fruits before and after the appearance of peel browning spots. Based on the overall results, it was speculated that the expressions of *CAD9* and *CCR* played a significant role before and after the development of peel browning spots. Furthermore, the unbagged and two-layer bagged fruits were consistent, and single-layer and triple-layer bagged fruits were consistent irrespective of the fruit color or the incidence of peel browning spots after bagging treatment. The rainy season occurs frequently during the ripening period of ‘Huangguan’ pears in mid–late July [1]. In this context, bagging enhances the temperature and humidity of the micro-domain environment within the bag. White two-layer paper bags had a higher transmission rate than brick-red single-layer paper bags and yellow triple-layer paper bags. Light has been shown to alter lignin production at the transcriptional level [36]. Therefore, the effects of light on the formation of peel browning spots on bagged ‘Huangguan’ pear require additional investigations.

## 4. Materials and Methods

### 4.1. Materials and Treatment

The experiment was conducted in the Fan Zhuang Orchard, Zhao County (37.756498° N, 114.776187° E), Hebei Province in 2017. Regarding climate conditions, the average temperature ranged from 15 to 33 °C from May to August. It was hot and rainy from July to August; 68% of the local rainfall (about 340 mm) was concentrated in this period. Fruits from healthy 20-year-old ‘Huangguan’ pear trees, cultivated conventionally in the field, were bagged six weeks after full bloom (18 May). In this case, three bagging treatments were applied, namely a single-layer paper bag in brick red (light transmittance: about 10%), a two-layer paper bag with both the outer and inner layers in white (light transmittance: about 40%) and a triple-layer paper bag that was yellow on the outside, black in the middle and white on the inside (light transmittance: 0%) (the specifications for length were 185 mm, with a width of 155 mm. Zhao County Zhihui Fruit Bag Factory, Shijiazhuang, China). Non-bagged fruit served as the control. Each treatment was repeated three times with one tree for each replication.

Fruit sampling was performed every two weeks from week 5 to week 15 after full blooms and weekly after that until maturation (10 August). Fifteen fruits were collected from each treatment, and five of them were used to observe lenticels, peel structure and other indicators. The peels of the remaining ten fruits were snap-frozen in liquid nitrogen and stored at −80 °C to analyze the enzymatic activity and related gene expression. Meanwhile, another fifteen fruits from each treatment were selected to assess their quality indicators such as fruit weight, firmness, SSCs, TA, Vc content, and peel browning spot index.

### 4.2. Measurement Indexes and Methods

#### 4.2.1. Counting of Lenticels

Pan et al. [37] used the following method to count lenticels. In this case, the middle of a tissue paper was cut into a square cavity with side lengths of 1 cm. It was then used to cover the lenticels at the equator of the shady and sunny sides of the fruit. The lenticels were counted and marked with a magnifying glass before calculating the average number per cm^2^.

#### 4.2.2. Observation of Pericarp Structure

Semi-thin sections of the pericarp were prepared according to Aparici and Marsden’s method [38]. After cutting 0.5 cm × 0.5 cm pericarp pieces from five equally spaced surfaces in the midabdominal line of the fruit, the samples were placed into a 2 mL centrifuge tube filled with 2.5% glutaraldehyde. The samples were then stored in a vacuum at 4 °C. The centrifuge tubes were rinsed with phosphate buffer and deionized water successively. After that, 1% osmium tetroxide solution was added to the centrifuge tube and fixed at 4 °C for 3 h. Then, acetone was used to clean the centrifuge tubes, which had been washed with phosphate buffer solution, rinsed with double-distilled water, and dehydrated with an ethanol gradient for 20 min each. They were subsequently reduced after infiltrating with Spurr resin, polymerizing at 65 °C and embedded. The embedded blocks were cut into 1.75 µm thick sections on a freezing ultrathin microtome, placed on a slide dripped with DI water, and baked with an alcohol lamp until the water dried.

Lignin staining was performed according to the method of Alba et al. [39]. To the cut semi-thin section, 1% (W) of phloroglucinol solution was added dropwise, and this was followed by the dropwise addition of diluted concentrated hydrochloric acid (V_concentrated hydrochloric acid_: V_distilled water_ = 2:1) to the slide. After staining, the liquid on the slide was decanted and the latter was wiped with filter paper. For cuticle staining, Brundrett et al. [40] used this method. Sudan red solution was dropped on the semi-thin section containing the peel. After baking under an alcohol lamp, it was stained at room temperature. After staining, the slides were fire-roasted, rinsed with DI water, and wiped clean with filter paper.

At a magnification of 10x, stained sections were examined and photographed using an Olympus microscope. A minimum of 10 representative fields were selected to calculate the lignin area ratio. The cuticle thickness was measured in 20 fields using Image Tool 3.0 (University of Texas, Austin, TX, USA).

#### 4.2.3. Measurement of Quality Indicators

The weight of the fruit was measured up to the nearest one thousandth with an electronic balance (ACS-30-J, Senssun, Guangdong, China). Fruit weight was expressed as Kg.

Firmness was then measured with a fruit hardness tester (GY-4, Tuopu Instrument, Zhejiang, China), equipped with a 10 mm diameter probe. First, the fruit peel was removed, and then the penetrometer was pushed into the fruit flesh at a depth of 10 mm. Each fruit was measured on two opposite points on the equatorial line of the fruit, and the average value was considered the firmness of that fruit. Firmness was expressed as kg·cm^−2^.

After measuring the fruit’s firmness, the liquid was extracted using a pipette. The SSCs was calculated and expressed as a percentage (%) after measuring the juice’s refractive index with a portable brix analyzer (PAL-1, Atago, Japan).

With a knife, pulp was removed from the opposite side of the fruit, and processed into puree. Then, TA and Vc were determined using the acid–base titration method described by Cao et al. [41]. TA and Vc were expressed as % and mg·100 g^−1^, respectively.

#### 4.2.4. Determination of L, a, b and I_AD_

The parameters related to peel color were measured using a colorimeter (CR-400, Konica Minolta Sensing Inc., Tokyo, Japan) as described by McGuire [42]. In this case, the L value reflected the brightness of the pericarp. In addition, red represented a positive value for variable a, whereas green indicated a negative value. In the same manner, the color yellow represented a positive value for b, but the color blue denoted a negative one. The surface on the midventral line of the shady and sunny sides of the pear was selected for taking readings, with the colorimeter corrected with a standard white color before each measurement.

The reticle was calibrated at the equatorial position of each selected pear. As required by the DA-Meter’s instructions (patented product of Bologna University, Bologna, Italy), the probe was pressed against the peel of the fruit where a cross was painted. Then, variations in the peel’s chlorophyll absorbance (index of absorbance difference, or I_AD_) were used to determine the peel’s maturity. Before every test, the standard plate underwent a white calibration.

#### 4.2.5. Measurement of Lignin Content

The Bruce and West’s method was used to calculate the lignin content [43]. Peel samples (1 g) ground in liquid nitrogen were mixed with 5 mL of ethanol. The samples were then centrifuged at 12,000× *g* for 20 min at 4 °C, with the resulting residue allowed to dry overnight at 25 °C. To 50 mg of the dried sample, 5 mL of 2 mol/L HCl and 0.5 mL of *β*-thioacetic acid were added. The mixture was then heated at 100 °C for 8 h and cooled on ice before centrifugation at 12,000× *g* for 20 min at 4 °C. The resulting residue was washed with distilled water, suspended in 5 mL of 1 mol/L NaOH and slowly shaken at 25 °C for 18 h before another centrifugation at 12,000× *g* for 20 min. This time, the supernatant was retained, and 1 mL of concentrated HCl was added for precipitation at 4 °C for 4 h. After 20 min of centrifugation at 12,000× *g* and 4 °C, the final residue was dissolved in 1 mL of 1 mol/L NaOH for measuring absorbance values at 280 nm. For this experiment, a blank control was provided with NaOH solution. The lignin content was determined using lignin-sodium sulfonate to produce a standard curve.

#### 4.2.6. Measurement of Total Phenolic Content

The total phenolic content was determined according to the Folin–phenol method [44]. Briefly, after grinding frozen peel samples with liquid nitrogen, 0.5 g was weighed and dissolved in 5 mL of 80% ethanol (*v*/*v*). This was followed by ultrasound extraction in the dark after 15 min of centrifugation at 10,000× *g* and 4 °C. The resulting supernatant was collected to measure the absorbance value at a wavelength of 760 nm. A standard curve was generated using gallic acid, and the phenol content was then expressed as mg/g FW.

#### 4.2.7. Determination of Enzymatic Activity

Enzymatic activities were determined according to the methods of Cao et al. [41] and Wang et al. [23]. Samples of the pericarp (0.1 g) were ground in liquid nitrogen before adding 1 mL of extraction solutions for PAL, PPO or POD. The mixture was then centrifuged at 12,000× *g* for 30 min at 4 °C, with the resulting supernatant stored at 4 °C as crude enzyme extract.

Determination of PAL activity: The enzyme solution was added to tubes containing boric acid–borax buffer and L-alanine solution. In this case, enzymes inactivated by boiling were used as the control. Tubes containing the enzyme solution or the control were incubated at 37 °C for 1 h before adding 0.1 mL of 6 mol/L HCl solution to terminate the reaction. Subsequently, at 290 nanometers, absorbance values of the solutions were documented following a zeroing process utilizing distilled water. In this experiment, a unit of enzymatic activity was defined as the quantity of enzyme necessary to induce a 0.01 per hour change in absorbance.

Determination of PPO activity: The enzyme solution was added to a phosphate buffer containing 50 mmol/L of catechol, after which absorbance values were measured at 420 nm every 30 s for 3 min. The amount of enzyme required to change the absorbance reading by 0.01 per minute was taken as one unit of enzymatic activity. Distilled water was used as a control for this experiment.

Determination of POD activity: The enzyme solution was added to 25 mmol/L of guaiacol before adding 0.5 mol/L of H_2_O_2_. Absorbance values were then measured at 470 nm every 30 s for 3 min, with one unit of enzymatic activity defined as the amount of enzyme required for an absorbance change of 0.01 per minute. Distilled water was used as the control.

#### 4.2.8. RNA Extraction and Analysis of Gene Expression

In total, 100 mg of frozen peel was weighed and ground in liquid nitrogen for RNA extraction. Total RNA was then extracted with an RNAprep Pure Plant Kit (Tiangen Biotech, Beijing, China) following the manufacturer’s instructions. This was followed by reverse transcription of 500 ng of total RNA using the PrimeScript™ RT Reagent Kit with the gDNA Eraser (TaKaRa, Dalian, China) kit, with the resulting cDNA subsequently amplified on an ABI7500 real-time PCR system (Applied Biosystems Inc., Foster City, CA, USA) as required by the specifications of the SYBR^®^ Premix Ex Taq™ II Kit (TaKaRa, Dalian, China). The relative expression levels of *CAD8*, *CAD9*, *CCR3*, *CCR4*, *CCR5*, *4CL6* and *C4H1* were then calculated according to the 2^−∆∆*Ct*^ method [45], with pear *PbActin2* selected as the reference gene [46]. The primer sequences, shown in Table 2, were designed based on the genome of Dangshan pear in NCBI (http://www.ncbi.nlm.nih.gov/genome/12793, accessed on 25 April 2023) before being synthesized by Sangon Bioengineering (Shanghai, China) Co., Ltd.

### 4.3. Statistical Analysis

All experimental data were statistically analyzed by SPSS software (Version 18.0, SPSS Inc., Chicago, IL, USA), with the results presented as the mean ± SD of three replicates. Significant differences between means were analyzed using the one-way Duncan’s multiple range test (*p* < 0.05).

## 5. Conclusions

In this study, bagging improved the appearance and surface structure of the ‘Huangguan’ pears. The fruit’s surface became smooth, and the lenticels became smaller. The surfaces of unbagged and two-layer bagged fruits were yellow-green, while that of single- and triple-layer bagged fruits were yellow-white. The fruits in the triple-layer bagged arrangement had superior internal quality compared to the other groups. Nevertheless, it also had the highest incidence of peel browning spots. The triple-layer bagged fruit had the highest phenolic contents in its peel both before and after peel browning spots. Still, its lignin contents were significantly lower than the other groups. Furthermore, the activities of lignin synthesis-related enzymes PAL, POD and PPO were the lowest, with the expression of genes related to lignin synthesis (*CAD*, *CCR*, *4CL6*, and *C4H1*) also being the lowest. Therefore, lignin synthesis might affect the peel browning spots of bagged fruits, while POD activity alongside the expression of *CAD9*, *CCR3*, *CCR4*, and *CCR5* genes played an essential role in peel browning spots. In summary, it provides a theoretical foundation for the influence of bagging on the peel browning spots during the growth and development of the ‘Huangguan’ pear. In addition, growers in rainy areas should use triple-layer paper bags with caution.

## Figures and Tables

**Table 1:** The effects of different bagging treatments on the incidences of peel browning spots of ‘Huangguan’ pears.

Bag Type	Incidences of Peel Browning Spots (%)	
	Weeks after Bagging (Weeks after Full Bloom)	
	7 (13)	9 (15)
No bagging	0 ± 0.00 d	0 ± 0.00 d
Single-layer paper bag	0 ± 0.00 d	13.33 ± 0.06 b
Two-layer paper bag	0 ± 0.00 d	10.00 ± 0.00 c
Triple-layer paper bag	0 ± 0.00 d	66.67 ± 0.06 a

Note: Values are means ± SD (*n* = 3) and different letters in the same column indicate statistically significant differences at *p* < 0.05.

**Table 2:** Sequence of primers.

Genes	GenBank Accession No.	Forward Primer	Reverse Primer
*PbCAD8*	XM_009363237.2	5′-CCAAAAACTTAAAACTGTTCACCAA-3′	5′-ACTCGTCTGTGAGCTCATGC-3′
*PbCAD9*	XM_009363233.2	5′-GGGCACCAACCGAAGTTAAA-3′	5′-TGTTTTGTACCTCCCGCTGC-3′
*PbCCR3*	XM_009371295.2	5′-GGTAGGCACTTGTGTGTGGA-3′	5′-TGGCAAACTGGGAACCTTGT-3′
*PbCCR4*	XM_018651024.1	5′-CAAGGCTTGCAAAAAGGCCA-3′	5′-ACTCCGGGTTCATAACGACG-3′
*PbCCR5*	XM_009356071.2	5′-GCAGCAAATAATGGGCGCTT-3′	5′-CGACTTGGGATGTCGGACTG-3′
*Pb4CL6*	XM_009358640.2	5′-GCTTTATCGGGAAGGCTTGTC-3′	5′-AATGGCAGCACAGCTGAATATC-3′
*PbC4H1*	XM_009376113.2	5′-AACTTCGAGCTTCTGCCTCC-3′	5′-CCCCAAGCATCAATCTACGC-3′
*PbActin2*	GU830959	5′-GGACATTCAACCCCTCGTCT-3′	5′-ATCCTTCTGACCCATACCAACC-3′

## Data Availability

Data are available upon request.
